# Multi-parent populations in crops: a toolbox integrating genomics and genetic mapping with breeding

**DOI:** 10.1038/s41437-020-0336-6

**Published:** 2020-07-03

**Authors:** Michael F. Scott, Olufunmilayo Ladejobi, Samer Amer, Alison R. Bentley, Jay Biernaskie, Scott A. Boden, Matt Clark, Matteo Dell’Acqua, Laura E. Dixon, Carla V. Filippi, Nick Fradgley, Keith A. Gardner, Ian J. Mackay, Donal O’Sullivan, Lawrence Percival-Alwyn, Manish Roorkiwal, Rakesh Kumar Singh, Mahendar Thudi, Rajeev Kumar Varshney, Luca Venturini, Alex Whan, James Cockram, Richard Mott

**Affiliations:** 1 UCL Genetics Institute, Gower Street, London, WC1E 6BT UK; 2 University of Reading, Reading, RG6 6AH UK; 3 Faculty of Agriculture, Alexandria University, Alexandria, 23714 Egypt; 4 The John Bingham Laboratory, NIAB, 93 Lawrence Weaver Road, Cambridge, CB3 0LE UK; 5 Department of Plant Sciences, University of Oxford, South Parks Road, Oxford, OX1 3RB UK; 6 School of Agriculture, Food and Wine, University of Adelaide, Glen Osmond, SA 5064 Australia; 7 Natural History Museum, London, UK; 8 Institute of Life Sciences, Scuola Superiore Sant’Anna, Pisa, Italy; 9 Faculty of Biological Sciences, University of Leeds, Leeds, LS2 9JT UK; 10 Instituto de Agrobiotecnología y Biología Molecular (IABIMO), INTA-CONICET, Nicolas Repetto y Los Reseros s/n, 1686 Hurlingham, Buenos Aires Argentina; 11 SRUC, West Mains Road, Kings Buildings, Edinburgh, EH9 3JG UK; 12 Center of Excellence in Genomics and Systems Biology, International Crops Research Institute for the Semi-Arid Tropics (ICRISAT), Hyderabad, India; 13 International Center for Biosaline Agriculture, Academic City, Dubai, United Arab Emirates; 14 CSIRO, GPO Box 1700, Canberra, ACT 2601 Australia

**Keywords:** Plant genetics, Plant breeding, Agricultural genetics, Quantitative trait

## Abstract

Crop populations derived from experimental crosses enable the genetic dissection of complex traits and support modern plant breeding. Among these, multi-parent populations now play a central role. By mixing and recombining the genomes of multiple founders, multi-parent populations combine many commonly sought beneficial properties of genetic mapping populations. For example, they have high power and resolution for mapping quantitative trait loci, high genetic diversity and minimal population structure. Many multi-parent populations have been constructed in crop species, and their inbred germplasm and associated phenotypic and genotypic data serve as enduring resources. Their utility has grown from being a tool for mapping quantitative trait loci to a means of providing germplasm for breeding programmes. Genomics approaches, including de novo genome assemblies and gene annotations for the population founders, have allowed the imputation of rich sequence information into the descendent population, expanding the breadth of research and breeding applications of multi-parent populations. Here, we report recent successes from crop multi-parent populations in crops. We also propose an ideal genotypic, phenotypic and germplasm ‘package’ that multi-parent populations should feature to optimise their use as powerful community resources for crop research, development and breeding.

Over recent years, numerous multi-parent populations (MPPs) have been successfully developed in crops (Huang et al. 2015; Cockram and Mackay 2018). MPPs bring together key genomic, phenotypic and germplasm resources to form a platform for research and development. In this review, we examine three themes covering new developments in crop MPP research: (1) we survey the rapidly expanding variety of crop MPPs, explaining how differences in their design and construction affect their power and precision in mapping quantitative trait loci (QTL), on which we provide a brief primer. (2) We review the use of genomic technologies in MPPs, which have proven particularly suitable for gathering dense genomic information across large populations. We make general recommendations for collecting genotypic resources in MPPs. (3) We discuss successful applications of MPPs, particularly where they have been used for breeding and pre-breeding. This includes the identification of QTL, the application of genomic prediction to MPPs, and the direct use of MPP lines as germplasm for varietal release or pre-breeding. These recent developments have shown the potential of MPPs for crop improvement.

## Multi-parent populations (MPPs)

Bi-parental populations, derived from crosses between two inbred lines, have been the standard design for genetic mapping in crops. There are three key advantages to bi-parental populations: (1) the relative simplicity of their construction. Just two generations are needed for F_2_ (selfed/inter-crossed F_1_ hybrids) populations, and only about six further generations of inbreeding in self-fertilising species are needed to make recombinant inbred lines (RILs) whose genomes are fixed. (2) Their high power to detect QTL because all allele frequencies are typically close to the optimal value of 50%. (3) The low rate of linkage disequilibrium decay within chromosomes. There are normally only one or two recombinants per chromosome arm (inbreeding a RIL only adds about one observable recombinant per arm) meaning only a few hundred genotyped markers are needed to map QTL.

However, bi-parental populations have two principal disadvantages: the lack of mapping precision, which stems from limited effective recombination occurring during population development, and low genetic diversity, which is due to the genetic bottleneck caused by the choice of two founders. This may limit the number of QTL captured as no more than two alleles segregate at any locus. Consequently, around a decade ago, a second generation of experimental mapping populations, initially utilising additional crossing generations in a bi-parental but eventually inter-crossing multiple parents (MPPs), was developed to address these issues.

The limited genetic recombination in bi-parental populations was first addressed via the advanced inter-cross (AIC) design. These capture additional recombination via crossing the F_2_ generation for further generations prior to genetic mapping, effectively increasing the mapping precision (Dudley 1993; Darvasi and Soller 1995). Despite its potential benefits, AIC has seldom been used in crops. So far, examples of AIC exist in two plant species, thale cress (*Arabidopsis thaliana*, Gerald et al. 2014) and maize (*Zea mays*, Lee et al. 2002; Balint-Kurti et al. 2010), discussed further by Cockram and Mackay (2018). A possible reason for the lack of uptake, acknowledged by Darvasi and Soller (1995), is that simply increasing population size in bi-parental populations also increases mapping precision. Although large bi-parental populations also require increased phenotyping and genotyping, there is no requirement for additional crossing to create the population, which is particularly important for selfing species where manual crossing is onerous.

**Fig. 1:**
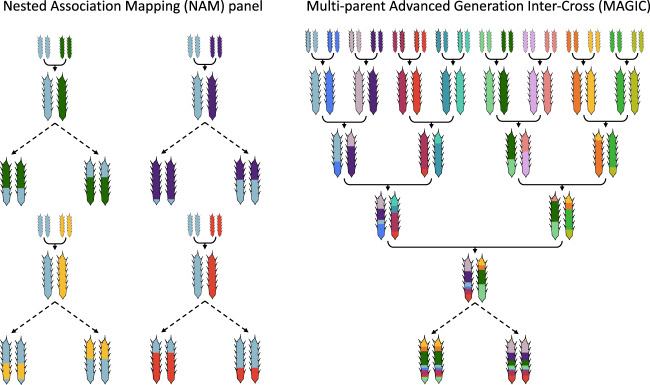
Common multi-parent population (MPP) designs. The nested association mapping (NAM) design consists of a series of bi-parental crosses against a common founder, from which recombinant inbred lines (RILs) are typically generated through selfing and single-seed descent (represented by dashed arrows). Hundreds of RILs can be derived from each bi-parental cross. Only four crosses are shown here but this design is readily extendable to include more founders. In the multi-parent advanced generation inter-cross (MAGIC) design, a series of equally balanced crosses are made between founders before RILs are developed.

Currently, the two most popular MPP designs in plants are nested association mapping (NAM) and multi-parent advanced generation inter-cross (MAGIC) populations. NAM population construction involves a series of crosses between a recurrent founder line and a number of alternative founders (Fig. 1). NAMs can be thought of as sets of bi-parental populations all linked by a common parent. They are therefore conceptually familiar for those used to working with bi-parental populations. While NAM captures additional genetic diversity, increased genetic recombination is essentially only captured via increasing the numbers of lines screened—as is the case in bi-parental populations. In contrast, the MAGIC design is more complex. MAGIC is an extension of AIC in some respects, except several founders are inter-crossed over multiple generations before selfing to generate inbred lines. MAGIC populations typically descend from 4, 8 or 16 parents, consistent with a simple funnel breeding design (Fig. 1; Huang et al. 2015). This is however not essential, for example, the first MAGIC population in plants used 19 *A. thaliana* parents (Kover et al. 2009). Each MAGIC line usually inherits alleles from all parents, and MAGIC chromosomes are random mosaics of the founder haplotypes. By capturing increased genetic recombination and genetic variation, MAGIC populations are designed to address both of the principal limitations of bi-parental populations for QTL mapping.

The beneficial properties of MPPs, namely high mapping power and resolution, expanded diversity compared with bi-parental populations, and their minimal population structure has increased their uptake in crop research. This increasing popularity of crop MPPs means that many now represent mature research and development tools. Most of the world’s major crops have spawned several MPPs (Table 1) and new MPPs for other crops are imminent (e.g., pigeonpea, *Cajanus cajan* and chickpea, *Cicer arietinum*, MAGIC populations with whole-genome sequence data, Pandey et al. 2016; Roorkiwal et al. 2020) or under development (e.g., sunflower, *Helianthus annuus*, Matias Dominguez, *personal communication* and *Triticum uratu*, MDA, *personal communication*). Where multiple MAGIC and/or NAM populations for the same crop are available, these are usually descended from different founders (Table 1). Availability of multiple MPPs offer the chance to replicate QTL across MPPs and to combine MPPs to improve power (Li et al. 2015).

**Table 1:** Examples of published plant multi-parent populations.

Population type	Species	Founders	Details	Genotype data	References
NAM	Barley (*Hordeum vulgare)*	26	25 wild barleys crossed to elite spring barley cultivar, 1420 RILs	5.709 genotyping array SNPs in 1420 RILs	(Maurer et al. 2015)
		26	25 wild barleys crossed to six-rowed spring malting barley cultivar (related to reference cultivar) and then backcrossed twice before RIL development, 769 RILs	384 SNPs assayed in 769 RILs; 4022 genotyping array SNPs and 263,531 variants from founder exome capture imputed into the population	(Nice et al. 2016)
		5	4 wild barleys crossed to reference cultivar, 916 RILs	384 SNPs assayed in 916 RILs	(Liller et al. 2017)
		89	88 six-row barley cultivars crossed to a six-rowed spring malting barley cultivar (related to reference cultivar), 6160 RILs	7773 genotyping array SNPs in 6160 RILs	(Hemshrot et al. 2019)
NAM	Bread wheat (*Triticum aestivum*)	11	9 Kenyan and 1 US spring wheats with stem rust resistance crossed against a Canadian spring wheat, 852 RILs	13.413 GBS SNPs in 852 RILs	(Bajgain et al. 2016)
		61	60 landraces crossed to elite spring wheat, reference line, 6240 RILs	2373 KASP SNPs and 33 SSRs on 2498 loci in 6240 RILs	(Wingen et al. 2017)
		29	25 global spring wheat landraces and 3 cultivars crossed to a broadly adapted cultivar, 2100 RILs	﻿57,657 genotyping array SNPs, ﻿﻿71,312 GBS SNPs and ﻿136,071 indels in 2100 RILs; founder exome sequences with ﻿638,045 SNPs and 63,022 insertions/deletions	(Jordan et al. 2018)
NAM	Durum wheat (*Triticum durum)*	37	50 Ethiopian farmer varieties crossed with an elite international variety, 6280 RILs	13,000 genotyping array SNPs for 100 RILs from each of 12 families	(Kidane et al. 2019)
NAM	Maize (*Zea mays*)	26	25 US inbred lines crossed to reference line, 6825 RILs	1536 genotyping array SNPs in 6825 RILs; up to 26 million SNPs imputed from ~4.3x founder WGS; ﻿294,962 GBS SNPs in ﻿4623 RILs; founder whole-genome assemblies available	(McMullen et al. 2009; Tian et al. 2011; Chia et al. 2012; Peiffer et al. 2014; Li et al. 2015) http://nam-genomes.org
		23	10 Dent founders crossed against a common Dent line and 11 Flint founders crossed against a common Flint line. Dent and Flint are major European maize germplasm pools. 2233 double haploid lines	﻿39,439 genotyping array SNPs in 2233 double haploid lines	(Bauer et al. 2013)
		12	11 Chinese inbred lines crossed to a common reference line, 2000 RILs	﻿238,945 GBS SNPs in 1696 RILs	(Li et al. 2015)
		6	4 *parviglumis* inbred teosinte lines and 1 *mexicana* teosinte inbred line crossed and backcrossed to a maize inbred line, 1257 RILs	51,544 GBS SNPs in 1257 RILs	(Chen et al. 2019)
NAM	Oilseed rape/Canola (*Brassica napus*)	16	15 semi-winter and spring inbred lines crossed to elite inbred variety, 2425 RILs	141,687 GBS SNPs in ﻿2425 RILs; ﻿﻿3,885,328 SNPs and 558,981 indels from founder WGS (10–18x) imputed in the population	(Schmutzer et al. 2015; Hu et al. 2018)
NAM	Peanut (*Arachis hypogaea*)	9	8 diverse lines crossed to 2 runner cultivars to create two NAM panels, ~6400 RILs	3874 genotyping array SNPs in 581 RILs in one NAM; 2680 genotyping array SNPs in 496 RILs in the other NAM	(Holbrook et al. 2013; Gangurde et al. 2019)
NAM	Rice (*Oryza sativa*)	11	10 tropical *japonica* lines crossed to elite *indica* reference line, 1879 RILs	70–85k GBS SNPs per family, most missing data imputed	(Fragoso et al. 2017)
NAM	Sorghum (*Sorghum bicolor*)	57	56 diverse lines backcrossed to a male-sterile version of an elite line, 4000 RILs	﻿932 DArT markers in 1389 RILs in 24 families	(Jordan et al. 2011; Mace et al. 2013)
		6	1 short, early elite line crossed and backcrossed to 5 ﻿tall, late, exotic lines, 724 lines	9139 GBS SNPs in 724 lines	(Higgins et al. 2014)
		11	10 diverse global lines to elite line, 2214 RILs	90,000 GBS SNPs in 2214 RILs	(Bouchet et al. 2017)
NAM	Soybean (*Glycene max*)	41	40 elite or diverse accessions to high yielding cultivar, 5600 RILs	4312 genotyping array SNPs in 5176 RILs	(Song et al. 2017; Xavier et al. 2018)
MAGIC	*Arabidopsis thaliana*	19	19 accessions crossed in 342 pairs, then randomly inter-crossed for four generations, 1026 RILs	1260 genotyping array SNPs in 527 RILs; annotated reference genomes and transcriptomes for founders; 0.3x WGS on 488 RILs	(Scarcelli et al. 2007; Kover et al. 2009; Gan et al. 2011; Imprialou et al. 2017)
		8	8 accessions crossed in 6 four-founder funnels, 532 RILs	91 SSRs and 230 SNPs in 532 RILs	(Huang et al. 2011)
MAGIC	Barley (*Hordeum vulgare)*	8	7 German landraces and 1 modern variety, crossed in 2 funnels, 533 doubled haploid lines	4.550 genotyping array SNPs in 533 doubled haploid lines	(Sannemann et al. 2015)
MAGIC	Bread wheat (*Triticum aestivum*)	4	4 elite Australian cultivars, crossed in 3 funnels, 1,579 RILs	826 DArT markers, 283 SNPs, and 53 microsatellites in 871 RILs	(Huang et al. 2012)
		60	1 male-sterile line crossed and backcrossed with 59 European and worldwide lines before 12 generations of random intermating, 1000 RILs	8,632 genotyping array SNPs in 380 RILs and 56 founders	(Thépot et al. 2014)
		8	8 winter wheat varieties selected by UK wheat breeders, crossed in 180 funnels, 1091 RILs	18,601 genotyping array SNPs in 643 RILs	(Mackay et al. 2014; Gardner et al. 2016)
		8	1 Danish and 7 German elite winter wheat lines, crossed in 2 funnels with one further round of inter-crossing, 516 RILs	5436 genotyping array SNPs in 394 RILs	(Stadlmeier et al. 2018)
		8	8 elite winter wheats with high German market share, crossed in 2 funnels, 910 RILs	7849 SNPs in 910 RILs	(Sannemann et al. 2018)
		8	3 Australian elite spring wheat cultivars, 4 worldwide spring wheat cultivars, and 1 Chinese winter wheat, crossed in 313 funnels followed by 0, 2 or 3 generations of inter-crossing, 3412 RILs	27,687 genotyping array SNPs	(Shah et al. 2019)
MAGIC	Chinese mustard (*Brassica juncea*)	8	8 founders chosen for agronomic traits, crossed in 1 funnel, 408 RILs	346 intron length polymorphism (ILP) markers in 113 RILs	(Yan et al. 2020)
MAGIC	Cotton (*Gossypium hirsutum*)	11	10 cultivars and one landrace crossed in 50 pairs, then randomly mated for 5 generations, 550 RILs in 55 families	6071 GBS SNPs and 223 microsatellite markers for 547 RILs; 473,517 SNPs from WGS founders at 20x and 550 RILs at 3x	(Islam et al. 2016; Naoumkina et al. 2019; Thyssen et al. 2019)
MAGIC	Cowpea (*Vigna unguiculata*)	8	1 US cultivar and 7 cultivars from sub-Saharan Africa chosen for agronomic traits, crossed in 6 funnels, 305 RILs	﻿32 130 genotyping array SNPs in 305 RILs	(Huynh et al. 2018)
MAGIC	Durum wheat (*Triticum durum)*	4	4 founders selected for diversity in origin and traits, crossed in 1 funnel, 338 RILs	7594 genotyping array SNPs in 338 RILs	(Milner et al. 2016)
MAGIC	Faba bean (*Vicia faba*)	11	11 European inbred lines, outcrossed for 9 generations, 189 RILs	156 SNPs assayed in 188 RILs	(Sallam and Martsch, 2015)
		4	4 inbred lines selected for geographic, genetic, and phenotypic diversity, crossed in one funnel, ~1200 RILs	To be genotyped using a genotyping array	(Khazaei et al. 2018)
MAGIC	Maize (*Zea mays*)	8	8 diverse inbred lines (+1 used to replace an incompatible two-way cross), crossed in 35 funnels, 1636 RILs	54,234k SNPs from WGS of founders (21–30x) imputed in 529 RILs; transcriptomes for 7 founders	(Dell’Acqua et al. 2015)
		4	4 inbred lines, crossed in 1 funnel, then 0, 1 or 2 rounds of inter-crossing before RIL development, 1005 RILs	﻿118,509 GBS SNPs and indels in 948 RILs	(Anderson et al. 2018; Mahan et al. 2018)
MAGIC	Rice (*Oryza sativa*)	8	8 elite *indica* varieties, crossed in 35 funnels, 2000 RILs	17,387 GBS SNPs for 200 RILs; 88,083 GBS SNPs for 1316 RILs	(Bandillo et al. 2013; Raghavan et al. 2017)
		8	Funnels from *indica* population above with two further rounds of inter-crossing before RIL development	14,242 GBS SNPs for 144 RILs	(Bandillo et al. 2013; Descalsota et al. 2018)
		8	8 elite *japonica* varieties, crossed in 35 funnels, 500 RILs		(Bandillo et al. 2013)
		16	150 inter-crosses made between funnels from the *indica* and *japonica* populations above, 1027 RILs	﻿66,309 GBS SNPs in 1027 RILs	(Bandillo et al. 2013; Zaw et al. 2019)
		12	12 breeder relevant lines, crossed in 2 funnels that share four founders between them, 206 RILs	86 SSR markers in 206 RILs	(Li et al. 2014)
		8	4 *indica* and four *japonica* elite cultivars from Japan, crossed in 2 funnels, 981 RILs	16,345 GBS SNPs in 981 RILs	(Ogawa et al. 2018)
		8	8 diverse *indica* lines from breeding programmes, crossed in 1 funnel, 1000 RILs with extra RILs developed from 4-way crosses	1329 genotyping array SNPs in 532 RILs (and 271 and 268 RILs derived from 4-way crosses)	(Meng et al. 2016)
		4	4 lines crossed in one funnel, 247 RILs	﻿843,505 SNPs from 30x WGS of the founders and 2x WGS of 247 RILs	(Han et al. 2020)
MAGIC	Sorghum (*Sorghum bicolor*)	29	10 male-sterile lines crossed with 19 lines chosen for agronomic traits, 9 rounds of random inter-crossing, 1000 RILs	79,728 GBS SNPs for 200 RILs	(Ongom and Ejeta 2018)
MAGIC	Strawberry (*Fragaria × ananassa*)	6	6 founders selected for genetic diversity crossed in 3 pairs then inter-crossed for 2 generations, 338 individuals	336 expressed sequence tag – simple sequence repeat (EST-SSR) in 338 lines	(Wada et al. 2017)
MAGIC	Tomato (*Solanum lycopersicum*)	8	﻿4 *S. lycopersicum* and 4 *S. lycopersicum var. cerasiforme* diverse lines, crossed in 1 funnel, 397 RILs	4 million SNPs from WGS of founders (6.7–16.6x); 1536 KASP SNPs genotyped in 397 RILs	(Causse et al. 2013; Pascual et al. 2015)

**Fig. 2:**
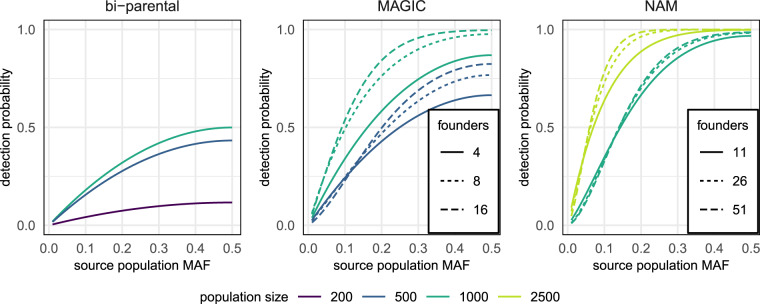
Probability of detection of a QTL within different experimental designs. The founders are assumed to be randomly selected from a source population in which a bi-allelic causal variant segregates at a particular minor allele frequency (MAF). Detection probability reflects both the number of founders that vary at the causal locus (calculated using binomial sampling) and the corresponding mapping power within the experimental population, which was calculated using a significance threshold of 10^−^^4^ and assuming that 5% of the phenotypic variation in a bi-parental population would be explained by the QTL: ${\it{\upbeta} }_{{\it{{\mathrm{QTL}}}}}^2 = 0.2$, ${\it{\upsigma }}^2 = {\it{\upsigma }}_{{\it{{\mathrm{QTL}}}}}^2 + 0.95$ see Eq. (1).

While this review focuses on NAM and MAGIC populations, many conceivable crossing designs can recombine multiple founders. Whatever the design, MPPs are generally: (a) derived from an explicit set of founders, preferably highly inbred lines or varieties that reproduce faithfully, (b) produced through experimental crosses that minimise selection and (c) composed of a large number of recombined individuals that are analysed together as a population. Philosophically, MPPs are established for long-term durability rather than short-term expediency. Bi-parental crosses are very efficient for mapping genetic variants that are known or suspected to vary between parents. MPPs are typically made with greater agnosticism about target traits and/or causal variants. Figure 2 demonstrates that large MPPs give a relatively good chance of detecting QTL that segregate in the population from which the founders were chosen. Thus, once constructed, MPPs provide enduring platforms on which it may be worth mapping non-target traits and/or investigating the genetic architecture of complex traits and the relationships between traits in the source population more generally.

### MPP founder selection

Founder selection is a key issue in MPP design because it determines the pool of genetic variation that segregates in the population. In Fig. 2, we assume that the founders are chosen randomly from a source population. However, many MPPs specifically aim to maximise the genetic diversity captured by the founders, which can be aided by genetic algorithms for founder selection (Ladejobi et al. 2016). In addition, different MPPs may target different source populations of interest, which is typically the case when multiple MPPs exist for the same crop. For example, some MPPs target diversity among elite lines from a particular region (Bandillo et al. 2013; Mackay et al. 2014; Sannemann et al. 2018; Kidane et al. 2019), while other MPPs include landraces and wild accessions (Kover et al. 2009; Maurer et al. 2015). In other cases, founders are selected with particular traits in mind, e.g., MAGIC rice populations focussed on heat and biotic stress resistance (Leung et al. 2015). Table 1 includes brief details of founder selection strategies implemented across various MPPs to date.

Diverse, trait-specific and region-specific MPPs are useful in different scenarios. In general, those MPPs developed to maximise segregating genetic variation are rich in novel allelic combinations from the diverse founders. This makes them a permanent resource to analyse the genetic basis of complex traits in different environments. However, diverse MPPs are potentially less useful for the direct use of their germplasm in pre-breeding because undesirable alleles may segregate in the progeny (Huang et al. 2015). In contrast, MPPs based on a more conservative founder selection strategy optimised around particular varieties, traits or environments might be more quickly translated into superior breeding lines and are of greater immediate value to breeders. An intermediate MPP design may employ a mix of improved and adapted breeding lines together with diverse varieties or varieties for high yield, disease resistance and tolerance to abiotic stresses. When an MPP produces lines that combine several desirable traits, they can be used directly in breeding programmes (Descalsota et al. 2018; Zaw et al. 2019).

In the case of NAM populations, the use of a single recurrent founder that will be represented in 50% of the genomes of the resulting RILs requires particular attention. The recurrent founder may be chosen to be a standard reference variety. For example, a variety with a sequenced genome was chosen in maize (McMullen et al. 2009). If the population is designed to be used for pre-breeding material, it is key that the recurrent founder has good agronomic performance. For example, the Ethiopian durum wheat NAM was designed to mix diverse Ethiopian landraces with an elite international variety (Kidane et al. 2019). To reduce the representation of potentially maladaptive exotic genetic material in the population, backcrossing to an elite recurrent founder is also sometimes performed prior to RIL development (Jordan et al. 2011; Nice et al. 2016; Chen et al. 2019).

### Crossing design

Though sharing the same overall objective of increasing genetic diversity, NAM and MAGIC populations differ in the genetic features of their constituent RILs. Each MAGIC chromosome is a random mosaic of all the founder genomes, while a NAM chromosome is a mosaic of just the two parents in its family.

The interconnected design of NAM populations allows new families to be added incrementally. Some NAM populations capture genetic diversity from up to 90 founders and may include thousands of individual RILs (McMullen et al. 2009; Maurer et al. 2015; Bouchet et al. 2017; Kidane et al. 2019). However, although the overall diversity can be large, the recurrent founder in NAM limits the haplotypic diversity within any family of RILs (Ladejobi et al. 2016). Furthermore, the benefits of increasing genetic diversity by adding founders may need to be balanced against the number of RILs developed from each cross (Gage et al. 2020; Garin et al. 2020). We also note that tailored mapping methods may be required to account for differing recombination frequencies among the NAM’s constituent bi-parental families (Li et al. 2011).

MAGIC designs require several intermating generations, proportional to the logarithm of the number of founders (Huang et al. 2015). In addition, the founders can be brought together in many different ways, where a particular order of crossing is referred to as a ‘funnel’. For example, in a population with four founders—*A*, *B*, *C* and *D*—a line derived from [(*A* × *B*) × (*C* × *D*)] has come through a different funnel to one derived from [(*A* × *C*) × (*B* × *D*)]. The number of possible funnels is often large: a population with 8 founders has 315 possible funnels if reciprocal crosses are ignored (Mackay et al. 2014). When using fully inbred founders, the genotypes of the initial two-way cross combinations are fixed, but the subsequent four-way crosses are heterozygous with unknown genetic makeup, and alleles can be lost to sampling. Thus, replicates of each crossing combination should ideally be made for all subsequent intermating generations to capture independent recombination events. However, the large number of crosses required in order to make and replicate all funnels may be a considerable challenge to population construction.

Different approaches have been employed to minimise the crossing effort required to construct MAGIC populations. Most MAGIC populations use a much reduced subset of all possible funnels (see Table 1) and derive multiple RILs from each funnel. Stadlmeier et al. (2018) found that a small number of funnels with an extra generation of inter-crossing could capture recombination as effectively as a more comprehensive MAGIC design with all possible four-way crosses. Other designs reduce the crossing effort by using a subset of founders in each funnel (Huang et al. 2011; Li et al. 2014) or exploiting several generations of random mating (Scarcelli et al. 2007; Sallam and Martsch 2015; Islam et al. 2016; Wada et al. 2017). In sorghum and bread wheat, outcrossing for random mating has been enforced using male-sterile lines (Thépot et al. 2014; Ongom and Ejeta 2018). Of potential relevance to crop MPPs, in fruit flies (*Drosophila melanogaster*), interconnected MAGIC populations have been developed that expand diversity while maintaining the closed MAGIC mating design (King et al. 2012). This approach could be adopted in crop MAGIC populations that share founders (e.g., Huang et al. 2012; Shah et al. 2019) to combine desirable features from both NAM and MAGIC crossing designs.

A final comment on population structure in MPPs is required. RILs derived from the same MAGIC funnel or NAM family are more closely related than those from different MAGIC funnels or NAM families. This imposes a degree of population structure which could artificially inflate the significance of QTL if not controlled. Fortunately, there are now a suite of methods that largely eliminate the effects of unequal relatedness using linear mixed models (Bradbury et al. 2007; Kang et al. 2008; Zheng et al. 2015; Broman et al. 2019). These model the phenotypic covariance between individuals in terms of their genetic covariance, which is usually computed using correlations between marker dosages (Yang et al. 2011). In any event, population structure in experimental MPPs is generally much less pronounced than it is in populations derived from pre-existing germplasm.

### Power and precision in QTL mapping: a quick primer

We next summarise the key characteristics of MPP population designs, using theoretical results on power and recombination. There is a well-established general theory to calculate the power to detect a QTL segregating in any population (Lynch and Walsh 1998). Power is a function of the sample size *N* and the fraction $f = \sigma _{{\mathrm{QTL}}}^2/\sigma ^2$of the total phenotypic variance *σ*^2^ explained by the QTL, $\sigma _{{\mathrm{QTL}}}^2$. Specifically, the power is the probability that a chi-squared distribution with one degree of freedom and non-centrality parameter *Nf* exceeds *q*_0_, where *q*_0_ is the quantile corresponding to the genome-wide threshold *p* value for the test of the null hypothesis of no association at a locus (i.e., when the non-centrality parameter is equal to zero). Thus, power only depends on *Nf*: if the fraction of variance explained by a QTL, *f*, is halved but the sample size doubled then the power is unchanged. For inbred lines, the variance explained by a bi-allelic QTL with minor allele frequency *π* is

1 $${{\sigma }}_{{{{\mathrm{QTL}}}}}^2 = {{{\pi} }}(1 - {{\pi }}){{\beta }}_{{\it{{\mathrm{QTL}}}}}^2,$$

where ±$\beta _{{\mathrm{QTL}}}$ is the deviation from the mean phenotypic effect attributed to carrying either QTL allele. This formula is for inbred lines: it should be multiplied by 2 for F_2_ crosses. Questions of dominance do not arise in RILs because all loci should be homozygous. The formula neatly separates the underlying biological effect of an allele, $\beta _{{\mathrm{QTL}}}$, from the effect of allele frequency, *π*. Assuming that an allele has the same biological effect, $\beta _{{\mathrm{QTL}}}$, across genetic backgrounds, power is maximised when the allele frequency is half, as occurs for QTL that segregate in bi-parental populations. Reducing the minor allele frequency from 1/2 to 1/8, as occurs for alleles that are private to one founder in eight-parent MAGIC populations, will reduce power by 7/16 = 44%, so that sample size should be increased 2.3-fold to maintain power.

There is no equivalent general theory that gives a confidence interval for a QTL as a function of the sample size, QTL effect size, genetic map, etc. The most relevant result for MPPs is from Broman (2005), who derived formulae for the map expansion of MPPs assuming that 2^*n*^ founders are crossed together during the creation of each RIL (*n* = 1 for bi-parental and NAM populations). The probability $Q_n(r)$ that two neighbouring marker loci with recombination fraction *r* descend from the same founder in each RIL is

2 $${\it{Q}}_{\it{n}}({\it{r}}) = 1 - \frac{{(1 - {\it{r}})^{{\it{n}} - 1}}}{{(1 + 2{\it{r}})}}.$$

If the markers are very close together so *r* is small, then $Q_n(r) \approx \left( {n + 1} \right)r$. Thus, doubling the number of founders increases the probability of a recombinant by a linear amount. We expect, other things being equal, that a confidence interval for a 2^*n*^-parent MAGIC resembles that in a two-parent population except that it is scaled by $Q_1(r) / Q_n(r) \approx 2/(n + 1)$, which is 50% and 40% of the width of the 2-parent RIL, for 8- and 16-parent MAGIC populations, respectively. However, in practice other factors including allele frequencies and the sample size will change, and the confidence interval for a given QTL depends strongly on the local genetic map. Many QTL studies determine confidence intervals empirically using a LOD-drop approach, where the QTL extent includes all nearby markers that have *p* values of association that are within a set range compared with the strongest association. This has been shown to give reliable estimates of confidence intervals provided it is calibrated appropriately for each experimental population (Manichaikul et al. 2006). Empirical results also suggest that QTL effect size has an important influence on the interval size, with strong QTL better localised than weak QTL; thus, high power generally also implies shorter confidence intervals. A high density of recombinants around a weak QTL might not improve mapping resolution very much.

Both mapping power and resolution are expected to be roughly proportional to sample size. The mean total number of breakpoints between the markers with recombination fraction *r* in *N* individuals will be appoximately $N\left( {n + 1} \right)r$, so increasing the sample size to preserve power also increases the amount of recombination and therefore the mapping resolution. In NAMs, we note that only those families in which the QTL segregates are relevant for mapping resolution, so the effective value of *N* for recombination will be lower. However, NAMs are frequently larger than MAGIC populations, which preserves both mapping power and resolution in practice, at the expense of phenotyping and one-time genotyping effort (Dell’Acqua et al. 2015; Anderson et al. 2018).

## Application of genomics to MPPs

Next generation sequencing (NGS) platforms have revolutionised genetics by providing a genotyping technology which can in theory assay every base of an organism’s genome (Auton et al. 2015). As DNA sequencing throughput has increased, costs have dropped by several orders of magnitude (Mardis 2017). NGS-based genotyping has become a highly cost-effective and efficient agri-genomics tool in both model and non-model crop species including those with large and complex genomes (Cao et al. 2011; Cheng et al. 2019; Haberer et al. 2019; Lachagari et al. 2019). As NGS assembly algorithms and data types have improved, whole-genome sequencing (WGS) has been widely used for de novo assembly (Schatz et al. 2014; Zapata et al. 2016; Clavijo et al. 2017; IWGSC 2018; Haberer et al. 2019), as well as for re-sequencing, transcriptome sequencing (RNAseq) and epigenetic sequencing. When combined, these approaches allow experimental annotation of genes, their transcriptional variants and their transcriptional control (ENCODE Consortium 2012).

There are two main applications of NGS to MPPs. First, de novo assembly of reference genomes for each of the founders, where the development of improved DNA sequencing, algorithms and new data types now allow the construction of chromosome-level genome assemblies (Mascher et al. 2017). This permits the construction of a multiple sequence alignment of the founders, a generalisation of the pan-genome concept that focuses more on the presence/absence of the gene catalogue across the founders (Golicz et al. 2016; Gao et al. 2019), followed by a comprehensive re-annotation of the gene models in each founder. In Gan et al. (2011) it was shown that the gene models in the 19 founders of an *A. thaliana* MAGIC population were so divergent that a simple ‘lift-over’ of the reference annotation to each assembly caused dramatic and largely false over-prediction of deleterious mutations (e.g., premature stops and frame-shifts); RNAseq evidence showed that the effects of most of these mutations were skipped by subtle changes in splicing. Moreover, structural rearrangements revealed by de novo assembly may be significant. For example, gene expression may be perturbed by the change in regulatory context (Imprialou et al. 2017). These results demonstrate the promise of ongoing projects to produce genome assemblies for MPP founders in crop plants (e.g., https://nam-genomes.org, https://gtr.ukri.org/projects?ref=BB%2FP010741%2F1).

Second, NGS offers the ability to genotype new and existing markers. Common marker technologies such as single-nucleotide polymorphism (SNP) genotyping arrays and Kompetitive Allele Specific PCR (KASP) assays only assess known alleles at specifically designed loci and therefore tend to miss most rare variants, and even common alleles absent from the samples used to develop the assays (Burridge et al. 2018; You et al. 2018). In contrast, NGS technologies can be used to query either the whole genome (i.e., WGS) or a smaller, reproducible fraction thereof. This second strategy is referred to as reduced representation sequencing (RRS). RRS approaches include: hybridisation (e.g., exome capture, Parla et al. 2011), restriction-site-associated DNA sequencing (RAD-seq, Baird et al. 2008), double-digest RAD-seq (Peterson et al. 2012), genotyping-by-sequencing (Elshire et al. 2011) and diversity array technology-seq (Schouten et al. 2012). Whilst RRS approaches are cheap, it should be noted that sample preparation involves a greater number of steps than WGS and so introduce a higher degree of bias, which often results in missing data. In addition, the density of polymorphic markers will be lower when coding regions are targeted because they tend to be less variable.

Sparse genotyping can limit both the power and precision of QTL mapping. Equation (1) assumes that the causal variant has been genotyped, or that a surrogate marker in perfect linkage with it has. If this assumption is violated, the variance explained by the QTL is reduced by *ρ*^2^, the squared Pearson correlation between the genotyped marker and the causal variant, reducing power accordingly. Furthermore, Eq. (2) assumes that recombination breakpoints are observable. Wherever founders are identical, at least at the genotyped markers, their recombinants are invisible, limiting mapping resolution. For example, in wheat MAGIC populations, only 50–72% of the predicted recombination events appear to have been observable, possibly because the density of markers on genotyping arrays is insufficient to distinguish between founder haplotypes (Gardner et al. 2016; Stadlmeier et al. 2018). Finally, ascertainment bias in genotyping array design can make it particularly difficult to distinguish between haplotypes from diverse founders (Dell’Acqua et al. 2015).

Dense genomic information, in contrast, can identify alleles within QTL that are putatively causal. For example, Imprialou et al. (2017) used WGS to associate signatures of structural variation with phenotypic variation, finding a deletion containing three genes within a QTL for germination time in *A. thaliana*. Similarly, transcriptomic data can prioritise candidate genes. For example, Dell’Acqua et al. (2015) narrowed functional candidate genes in maize to those that had expression patterns in the founders consistent with the phenotypic effects that were estimated during QTL mapping.

### Imputation in MPPs: the power of haplotypes

A simple mapping strategy is to genotype and phenotype an MPP and then perform association mapping to identify QTL. While this can be successful, there are advantages in exploiting the fact that RIL chromosomes are recombination mosaics of the genomes of the founders. These mosaics can be inferred from SNPs, for example using a Hidden Markov Model (Mott et al. 2000; Liu et al. 2010; Zheng et al. 2015; Broman et al. 2019). In a bi-parental cross between inbred lines, bi-allelic polymorphic markers are sufficient to identify the parental origin of each RIL genomic locus. When there are multiple haplotypes and/or founders segregating in the populations, bi-allelic markers are not completely informative anymore and the haplotypic context given by surrounding markers is used to infer the founder that contributed each genomic locus within each RIL (Mott et al. 2000).

**Fig. 3:**
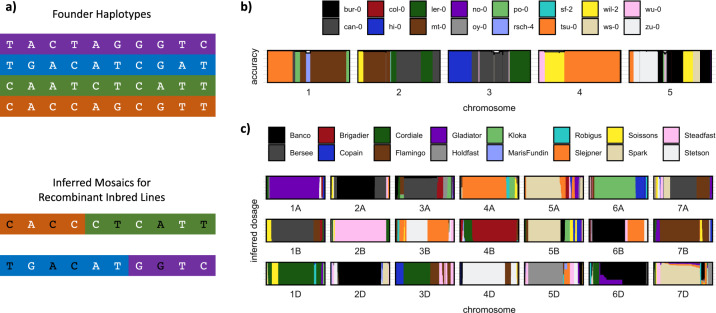
Reconstruction of MAGIC recombination mosaics and imputation. **a** In this schematic example, the MAGIC founders are genotyped more densely and confidently than the MAGIC recombinant inbred lines. The observed genotypes in the recombinant inbred lines (white) can be used to infer the ancestry mosaics (background colours) from which unobserved genotypes (black) can be imputed. Two examples of ancestry mosaics reconstructed from low-coverage sequence data in **b** *Arabidopsis thaliana* and **c** bread wheat. In **b**, the ancestry mosaic is estimated using the Reconstruction program (http://mtweb.cs.ucl.ac.uk/mus/www/19genomes/MAGICseq.htm), and accuracy is assessed as the fraction of mismatches in each block between the inferred founder haplotypes and calls derived directly from low-coverage sequencing data. In **c**, the inferred ancestry proportion probabilities (dosages) are emitted after imputation using the software, STITCH (Davies et al. 2016).

Once recombination mosaics have been constructed, sequence information from the founders can be projected onto RILs. Importantly, variants can be imputed even when they are not directly genotyped in any of the RILs. Thus, low-cost sparse genotyping data for the RILs can be used to infer the mosaics and then dense genomic data from the founders can be copied onto them to impute all variants (Fig. 3). In general, there is uncertainty in the genome mosaics and in the founder genomes, which can be easily accommodated by representing genotypes and haplotypes as dosages. Association mapping itself can be performed on the dosages of either haplotypes or imputed variants. Performing both types of association may be used to distinguish whether a QTL is caused by a bi-allelic variant or is haplotypic (Yalcin et al. 2005): evidence from an outbred eight-parent MPP in rats, *Rattus rattus*, suggests that ~40% of QTL are caused by multiple causal variants, or equivalently that multiple founder haplotypes have different effects (Baud et al. 2013).

Genotype imputation has been an active area of research in the past decade, and many powerful methods have been published and extensively applied already. Some of the most popular for human population studies are Beagle (Browning 2008) and IMPUTE (Howie et al. 2009). A common drawback of these established methods is that they rely on high sequencing depth and on the availability of a high-quality reference panel to infer and follow the haplotypes in the progeny. These conditions are, however, not always achieved for non-model organisms including crop species. For this reason, various methods have been developed to cater for the crop community, including mpMap (Huang et al. 2014), FILLIN (Swarts et al. 2014), STITCH (Davies et al. 2016), LB-Impute (Fragoso et al. 2016), LinkImputeR (Money et al. 2017) and magicImpute (Zheng et al. 2018). We note that the founder haplotypes of an MPP play a similar role to haplotype reference panels that are used in human genetics. However, while the latter is a construct, being simply a convenient set of basis haplotypes from which every chromosome can be derived as a mosaic, the MPP founder haplotypes are the real ancestral genomes for the MPP population and usually correspond to stable lines or varieties.

Imputation methods suggest a simple and cost-effective genotyping strategy: low-coverage (e.g., 0.3x–1.0x) WGS of each RIL. For example, using 476 *A. thaliana* MAGIC RILs sequenced at 0.5x, it was possible to impute SNPs with a concordance of 98% at sites previously genotyped by a conventional genotyping array (Imprialou et al. 2017). Within each constituent genome the coverage of each variable site will be random, and most sites will be covered by no more than one read. Nevertheless, by combining all the data from hundreds of RILs in a single analysis it is possible to simultaneously infer both the haplotype space and the mosaic of each chromosome in terms of these haplotypes, and hence impute the complete genome of each, even without knowledge of the founder genomes (Davies et al. 2016). It can be useful to infer founder haplotypes because unknown founders may have accidentally contributed to the population and/or the founders used for crossing may differ slightly from those sequenced. Nevertheless, the founders should ideally be characterised using orthogonal and more comprehensive genomic data; re-annotated de novo assemblies of the founders are the gold-standard. Given that researchers have to balance effort and resources, it’s significant that imputation in MPPs allows dense genomic information from a relatively small number of founder genomes to be leveraged across the population. Thus, MPPs provide one of the most cost-effective ways of obtaining near complete genomic information for a large genetically diverse population.

## Applications of MPPs

A primary use of crop MPPs is to map QTL for agronomic traits to relatively narrow genetic intervals across genetically diverse backgrounds. To date, many traits have been mapped in MPPs, ranging from pathogen resistance to flavour profile. Table 2 summarises the traits mapped just in crop MAGIC populations, encompassing simple to complex traits and exemplifying their utility in dissecting the genetic architecture of crop phenotypes. For example, 75–80% of the phenotypic variance in leaf length, width and angle in a large maize NAM population can be accounted for by more than 30 QTL that have been identified (Tian et al. 2011; Gage et al. 2020).

**Table 2:** Traits mapped in crop multi-parent advanced generation inter-cross (MAGIC) populations.

Crop	QTL mapping trait/s	Reference
Barley	Flowering time	(Sannemann et al. 2015; Mathew et al. 2018; Afsharyan et al. 2020)
Bread wheat	Plant height, hectolitre grain weight	(Huang et al. 2012)
	Awn presence/absence	(Mackay et al. 2014)
	SnTox 1 and SnTox3 sensitivity (*Parastagonospora nodorum* fungal effectors)	(Cockram et al. 2015; Downie et al. 2018)
	ToxB sensitivity (*Pyrenophora tritici-repentis* fungal effector)	(Corsi et al. 2020)
	Coleoptile and shoot length	(Rebetzke et al. 2014)
	Grain dormancy	(Barrero et al. 2015)
	Inflorescence architecture and paired spikelet development	(Boden et al. 2015)
	Digitally extracted phenology and senescence	(Camargo et al. 2016)
	Powdery mildew resistance (seedling)	(Stadlmeier et al. 2018)
	Plant height	(Sannemann et al. 2018)
	Inflorescence architecture and development	(Dixon et al. 2018)
	Digitally extracted plant area, height, water use and senescence	(Camargo et al. 2018)
	Powdery mildew, *Septoria tritici* blotch and tan spot disease resistance	(Stadlmeier et al. 2019)
	*Septoria nodorum* blotch disease resistance (in leaf and glume tissues)	(Lin et al. 2020)
Chinese mustard (*Brassica juncea*)	Glucosinolate content	(Yan et al. 2020)
Cotton	Fibre quality	(Islam et al. 2016; Thyssen et al. 2019)
	Fibre length	(Naoumkina et al. 2019)
	Root-knot nematode resistance	(Wubben et al. 2019)
	Fibre maturity and fineness	(Kim et al. 2020)
	Verticillium wilt resistance	(Zhang et al. 2020)
Cowpea	Flowering time, growth habit, flower colour, leaf shape and seed characteristics	(Huynh et al. 2018)
Durum wheat	Grain yield	(Milner et al. 2016)
Faba bean	Frost tolerance and related traits	(Sallam and Martsch 2015)
Maize	Grain yield, flowering time	(Dell’Acqua et al. 2015)
	Genetic recombination	(Guan et al. 2017)
	﻿Plant height, ear height and flowering time	(Anderson et al. 2018)
	Corn borer resistance	(Jiménez-Galindo et al. 2019)
	Fusarium seedling rot resistance	(Septiani et al. 2019)
	Fusarium ear rot resistance	(Butrón et al. 2019)
	Stover yield and saccharification efficiency	(Lopez-Malvar et al. 2020)
	Cold tolerance	(Yi et al. 2020)
Rice	Blast/bacterial blight resistance, salinity/submergence tolerance and grain quality	(Bandillo et al. 2013)
	Yield and yield components	(Meng, Zhao et al. 2016)
	Plant height, heading date	(Meng, Guo et al. 2016)
	Yield and yield components, flowering time, plant height, amylose content, submergence tolerance and brown spot disease resistance	(Raghavan et al. 2017)
	Yield and yield components, plant height, bacterial leaf blight, flowering time and biofortification	(Descalsota et al. 2018)
	Bacterial leaf streak and bacterial leaf blight	(Bossa-Castro et al. 2018)
	Grain shape	(Ogawa, Nonoue et al. 2018)
	Grain quality, cooking and taste attributes	(Ponce et al. 2018)
	Yield, plant height, heading date, grain quality and biofortification	(Zaw et al. 2019)
	Grain length, grain width, grain thickness, and thousand grain weight	(Ponce et al. 2020)
	Heading date	(Han et al. 2020)
Sorghum	Plant height	(Ongom and Ejeta 2018)
Strawberry	Fruit quality-related traits	(Wada et al. 2017)
Tomato	Fruit weight	(Pascual et al. 2015)
	Fruit weight and flowering time plasticity in response to water, salinity and heat stress	(Diouf et al. 2020)

One purpose of mapping QTL is to understand the genetic basis of traits and to identify markers that can be used in breeding programmes. In applied settings, key factors influencing whether QTL are useful are the size and stability of the effect across environments and genetic backgrounds, and the phenotyping effort required to assess the trait directly. While causal markers reduce the potential for linkage drag (introgression of linked but unfavourable alleles) during marker-assisted selection, nearby markers that tag the causal allele can be used instead. Similarly, studies of general patterns of genetic architecture do not require gene-level QTL mapping. For other theoretical and some applied applications (e.g., gene editing), validation of causal variants may be required. Thus, the research goals should generally determine the appropriate experimental design (e.g., population size and founder selection) and analysis (e.g., acceptable false positive rate), as reviewed elsewhere (Bernardo 2008, 2016).

In principle, all QTL identified from MPPs could have been mapped to the same accuracy in an appropriately chosen bi-parental population and/or a Genome-Wide Association Study (GWAS) that uses a panel of existing lines. As noted above, relative to bi-parental populations, MPPs are a more general tool within which a wider variety of traits segregate due to the increased genetic variation (Fig. 2). Compared with GWAS of pre-existing lines, experimental populations like MPPs largely avoid the potentially confounding influence of population structure and raise the allele frequency of a subset of alleles that are rare in the wider population. Thus, MPPs increase the overall probability of detecting very rare beneficial alleles. Such alleles are of particular interest for breeding (Bernardo 2016) but are difficult to detect, even in MPPs (Fig. 2). A further consideration is mapping resolution, which should be smallest in large GWAS that capture historical/natural recombination across the pre-existing germplasm, e.g., linkage decays to background levels over ~10 Kbp in wild *A. thaliana* accessions (Kim et al. 2007). In MAGIC populations of 527–529 RILs, QTL for various traits were mapped to intervals of 0.3–6 Mbp in *A. thaliana* (Kover et al. 2009) and 1.5–17 Mbp in maize (Dell’Acqua et al. 2015). In a MAGIC rice population of over 1316 RILs, the mapping intervals were ~700 Kbp on average (Raghavan et al. 2017). However, as discussed above, empirical mapping resolution may be determined by the QTL effect size and population size.

The wheat MAGIC populations developed at CSIRO (four Australian spring wheat founders, Huang et al. 2012) and NIAB (eight UK winter wheat founders, Mackay et al. 2014) are examples of MPPs that have been used to map genes controlling important yield related traits. The CSIRO spring wheat population was used to identify 18 QTL that influence the formation of paired spikelets—a modified form of inflorescence architecture in wheat. Due to the high density of polymorphic markers, the *Photoperiod-1* gene could be identified as a key regulator of paired spikelet development (Boden et al. 2015). Subsequent analysis of a RIL from the same population that robustly formed paired spikelets permitted dissection of a second QTL on chromosome 4D through investigation of a RIL-specific chromosomal duplication event of 4D, which doubled the copy number of *TEOSINTE BRANCHED 1* (*TB1*) (along with many other genes) (Dixon et al. 2018). That is, an unexpected duplication occurred during population development. The chromosomal duplication proved to be highly relevant when determining the causal gene between two closely located candidates: *TB1* and the Green Revolution gene, *Reduced height-1* (*Rht-1*). Analysis of spikelet architecture in the UK winter wheat eight-way population enabled identification of further allelic diversity for *TB1*. Mapping using this population demonstrated that the control of the paired spikelet formation by *TB1* also occurred in winter wheat and allowed identification of a new allele of *TB1* involved in the regulation of paired spikelet formation and height on chromosome 4B (*TB-B1*) (Dixon et al. 2018, 2020). Markers for *TB1* alleles are now used for marker-assisted selection in the wheat breeding industry.

After primary mapping, QTL are often fine mapped through further crosses (Jaganathan et al. 2020). In MPPs, the intermediate crosses made during population development can be useful because, at each stage, some lines may be heterozygous for alleles at a QTL of interest. These can be used to rapidly develop pairs of near isogenic lines (NILs) differing only at one or two haplotype blocks underlying the QTL of interest. NILs and/or RILs can then be used for further molecular characterization, validating candidate genes. For example, Liller et al. (2017) used NILs to narrow down a QTL for barley awns to a region containing 66 genes and used RNA-expression data to suggest two candidate genes; Wubben et al. (2019) used virus-induced gene silencing of four candidate genes in five MAGIC RILs to identify one that was required for root-knot nematode resistance in cotton.

Separate from direct applications in breeding, QTL mapping is used by evolutionary biologists to understand the genetic basis of adaptation. For this purpose, MPPs can be used to quickly dissect the genetic basis for variation in putatively adaptive traits. For example, the *A. thaliana* MAGIC population has been used to fine-map several traits involved in developmental timing (e.g., time to bolting, Kover et al. 2009) and to examine the natural genetic basis of variation in seed size and number (Gnan et al. 2014). In addition to examining natural variation, MPPs could be used to identify genes under selection in experimentally evolving populations. Using wheat, for example, experimental evolution has identified natural selection for key phenology genes at contrasting geographies in France (Rhoné et al. 2008). In another application, Knapp et al. (2020) found that several genes controlling plant height and phenology reverted to the wild type in experimental wheat populations evolving under natural selection in the UK. Similar approaches could exploit the genetic variation in crop MPPs to identify additional genes underlying environmental adaptation and historical selection. For example, using MPPs with founders that span early and modern agriculture, it should be possible to examine the evolutionary history of crop improvement, identifying traits and genes involved in historical yield increases. This in turn could provide valuable insights into the potential for future yield increases.

### Multi-trait analyses

Phenotyping information may be accumulated and shared in large and stable collections of inbred lines, particularly in crop MPPs. They are a convenient system in which to study interactions and correlations between traits—sometimes across multiple environments—and the extent to which their genetic basis is shared. Exploitation of MPPs to study trait–trait interactions at the level of the underlying genetics is one of their great, yet largely unfulfilled, applications. As high-throughput, high frequency phenotyping platforms become available, publicly accessible repositories of phenotypic data for multiple traits and environments will further enhance these applications.

As phenotypes for related traits are collected, multi-trait QTL analyses can be conducted (van Eeuwijk et al. 2010). Where data is incomplete, packages are available to impute missing phenotypes (Dahl et al. 2016). If multiple traits are measured, each with their own errors, but are under pleiotropic control of a single QTL, a combined analysis generalised across traits should give a more accurate indication of the true genetic effect. This has readily been applied in simple mapping populations (Hackett et al. 2001) and methods to analyse multi-trait and multi-environment traits in MPPs have been developed by Verbyla et al. (2014), allowing pleiotropic QTL and closely-linked QTL to be distinguished. Scutari et al. (2014), Descalsota et al. (2018) and Zaw et al. (2019) used Bayesian networks to simultaneously model multiple traits. Mapping composite traits derived from groups of correlated traits might uncover novel QTL that are not found when traits are analysed independently. Such analyses might suggest previously untried breeding routes to minimise trade-offs, for example, to avoid increasing yield at the expense of quality or delayed flowering, which increases the risk of encountering terminal drought (drought during grain filling) or adverse weather conditions at harvest that cause lodging or pre-harvest sprouting.

The accumulation of MPP trait data from multiple environments also enables QTL mapping of genotype by environment interactions, with the potential to breed for improved crop resilience to environmental stresses, and to understand and exploit the changes in trade-offs that occur under different environmental conditions. For example, the adaptation of *A. thaliana* to different environments has been studied in a MAGIC population. Several QTL were found to promote a plant’s fitness (reproductive success) in its native environment (Sweden or Italy) but reduce its fitness when transplanted to the non-native environment—a type of evolutionary trade-off (Ågren et al. 2013). In a tomato MAGIC population, trials across water, salinity and heat stress treatments revealed QTL that affect plasticity of response (Diouf et al. 2020). Garin et al. (2020) recently developed specific methods for multi-environment QTL analysis in MPPs and applied them to a European maize NAM population, finding alleles that have a greater effect on yield at sites with higher precipitation. This type of multi-environment trial analysis is also at the core of a rice MAGIC population constructed from founders that vary in their tolerance to temperature stresses (Leung et al. 2015).

Even ignoring genetic information, MPPs are valuable for dissecting correlations between phenotypes because they capture diversity and largely eliminate confounding population structure. As an illustrative example, consider plant height and root architecture in wheat. The height of wheat varieties has greatly decreased over the past century, particularly due to the introgression of large effect ‘Green Revolution’ alleles at the *Rht* genes that reduce the risk of yield loss through lodging (Hedden 2003). In addition, more-modern varieties tend to have smaller root systems, which may have been selected to increase yield by reducing below-ground competition within the crop (Fradgley et al. 2020). These traits (plant height and root architecture) may have a physiological connection causing them to be correlated. Alternatively, they may be correlated because modern varieties are likely to have experienced selection for both traits at independent loci. MPPs offer an opportunity to identify correlations between traits that are truly caused by a shared underlying genetic basis. For example, in a maize NAM, leaf length, width and angle have weak correlations (0.03–0.08) and share only 2–6% of QTL (Tian et al. 2011) whereas different carbon and nitrogen metabolites are more highly correlated (up to 0.7) and share QTL (Zhang et al. 2015; Gage et al. 2020).

### MPP germplasm in breeding programmes

Independent of QTL analysis, MPPs provide useful germplasm for breeding or pre-breeding activities. The extensive shuffling of genetic variation during population development generates novel allelic combinations. Therefore, a subset of lines will usually display better phenotypes than any of the parental lines. These ‘transgressive’ lines may be good breeding material in their own right (Huynh et al. 2018). This is particularly true when the parents are commercial/cultivated varieties, although the MPP may have taken several years to construct since the parent varieties were released. Several MPPs employ participatory methods in the prioritization and/or selection of founders and traits (e.g., Kidane et al. 2017, 2019; Mancini et al. 2017; Campanelli et al. 2019) to facilitate end-user applications.

There are several examples where MAGIC germplasm has been used for pre-breeding or released in their own right as a variety. Li et al. (2013, 2014) report that a RIL from a rice MAGIC population was released as a new variety in China. Separate rice MAGIC populations developed at the International Rice Research Institute (IRRI) have also been used; the MAGIC RIL, IR 95099:7-B-2-10-10-2, is in the pipeline of varietal release in southern Vietnam on the basis of its maturation date, yield and grain quality, which were assessed in trials at the Cuu Long Delta Rice Research Institute, Can Tho (RKS, *personal communication*). In chickpea, RILs from MAGIC populations developed at the International Crops Research Institute for the Semi-Arid Tropics (ICRISAT) have been directly released as new varieties, as well as used as donors in commercial breeding programmes (RKV, *personal communication*). From a 16-founder wheat MAGIC population developed at NIAB, 24 MAGIC RILs have been selected for inclusion in commercial breeding programmes on the basis of their yield, ear weight and protein content (NF, *personal communication*). Thus, the collection of phenotypic data across several agronomically important traits in MPPs facilitates the identification of promising lines.

A major impediment to the uptake of the results of QTL studies in breeding programmes is the crossing effort required to combine beneficial alleles at multiple loci (Bernardo 2008). In large and highly recombined MPPs, RILs that ‘pyramid’ several beneficial alleles will usually already exist. For example, the rice Bio-MAGIC population developed at IRRI demonstrates the pyramiding of multiple genes for three diseases (blast, bacterial blight and brown plant hopper) without employing backcrossing (Leung et al. 2015). Furthermore, Descalsota et al. (2018) and Zaw et al. (2019) identify rice MAGIC RILs with beneficial allelic combinations across grain yield, grain zinc content, flowering time, plant height and amylose content. Four lines carry tolerant alleles for multiple diseases and insects, as confirmed by re-sequencing data. These lines are planned to be used directly in breeding programmes (Hei Leung and RKS, *personal communication*). In cotton, Thyssen et al. (2019) identify MAGIC RILs that pyramid eight alleles with positive effects on four different measurements of fibre quality. Thus, the identification of QTL and their carriers—often part of initial population development and analysis—also primes MPP germplasm for agricultural use.

As largely unstructured populations with associated large-scale phenotype and genotype resources, MPPs are also highly suitable for genomic prediction. This process involves fitting a model to predict phenotypes from genotypes using a training dataset. The accuracy of this prediction can then be evaluated in a test set of lines. Prediction accuracies will be most accurate when the training set and test set are highly related, and more caution should be taken when applying predictions to more distantly related material. Bian and Holland (2017) reported reasonable within-family prediction ability for plant height, southern leaf blot resistance (prediction *R*^2^ ~ 0.5) and grey leaf spot resistance (*R*^2^ ~ 0.25) in a large maize NAM. Islam et al. (2020) used a MAGIC population of cotton to predict fibre quality traits across years, finding predictive abilities (correlation coefficients) ranging from 0.41 to 0.68. Although less accurate than direct phenotyping, genomic prediction can increase genetic gain by reducing the phenotyping time and effort required for selection after the model is trained. Zhang et al. (2017) implemented this type of selection regime in an MPP generated from two rounds of inter-crossing between 18 elite tropical maize lines, finding that realised yield increased by 0.1 tonnes per hectare per year with scope for further improvement using faster genotyping protocols. A potential drawback of genomic selection is that it may select against favourable contributions from exotic founders due to linkage drag, especially in MPPs with a mixture of elite and exotic founders. For this reason, Yang et al. (2019) demonstrate the use of origin-specific genomic selection in maize and barley NAM populations, suggesting a way to maximise genetic diversity for long-term genetic gain.

## The MPP ‘package’

MPPs integrate extensive genotype, phenotype and germplasm resources to provide enduring and general tools that advance theoretical knowledge and support breeding. MPP designs have allowed rich genomic information to be gathered for large populations in a cost-effective way. Furthermore, MPPs have proven to have useful applications in crop breeding. To fully realise their potential, we advocate thinking of MPPs as a package, ideally comprising:

(i) The germplasm of the RILs and their founders, free of intellectual property constraints.
(ii) A publicly accessible database of agronomically important phenotype data for the founders and the descendent RILs, with a system for adding further phenotypic data.
(iii) Complete de novo assemblies of the founders with catalogued genetic variation (including structural variation) and gene models confirmed using RNAseq.
(iv) The genome mosaics of the RILs, with founder annotations and variants projected and integrated software for mixed model GWAS at both SNP and haplotype level.
(v) Demonstration that the population can be used for genomic prediction, the identification of likely causal variants underlying QTL and marker development for marker-assisted selection.

Most crop MPPs currently only have a subset of these resources available. However, the continued enrichment of MPPs with open-access phenotypic and genotypic resources will enhance their power as an enduring and growing genetic toolbox to address crop improvement.
